# Bridging lived experience and research: involving dementia caregivers in developing interventions

**DOI:** 10.1002/alz70858_096506

**Published:** 2025-12-24

**Authors:** Frida Svedin, Ida Österman Menander, Oscar Blomberg, Anders Brantnell, Louise von Essen, Paul Farrand, Theresia Lückner, Kristina Sundelin, Jonna Turney, Anna Cristina Åberg, Joanne Woodford

**Affiliations:** ^1^ Uppsala University, Uppsala, Uppsala, Sweden; ^2^ University of Exeter, Exeter, Exeter, United Kingdom; ^3^ Dalarna University, Falun, Dalarna, Sweden

## Abstract

**Background:**

Public contribution in research during the intervention development phase can help inform the design of relevant, acceptable, and effective interventions. However, the experiences and impact of public contribution activities are seldom reported, particularly in the intervention development phase. We integrated public contribution activities throughout a series of studies to inform the development and adaptation of a psychological intervention for people with dementia and depression. Aims were to: (1) explore the experience, process, and perceived impact of involving informal caregivers as public contributors; (2) explore how to involve people with dementia and male caregivers in future public contribution activities.

**Method:**

A Public Advisory Group (PAG) consisting of wives and daughters (*n* = 4) of people with dementia was established to help: (1) make sense and interpret findings from the studies to inform the development and adaptation of the psychological intervention; and (2) co‐design the intervention. Public contribution activities were recorded using impact logs (*n* = 9). Based on impact logs, recommendations were extracted and percentages of those implemented were calculated. Upon completion of the intervention development phase, semi‐structured interviews were held with public contributors (*n* = 4) and researchers (*n* = 3) to explore their experiences and perceived impact of public contribution activities. Interviews were analyzed using manifest content analysis.

**Result:**

Public contributors made 158 recommendations across nine PAG meetings. In total, 76% of recommendations were implemented by the research team. Analysis of interviews generated three main categories: *Perceived impacts; Interactions and processes;* and *Future challenges and opportunities*. Interviews suggested that public contribution activities had a positive impact on the research e.g., by enhancing intervention acceptability and relevancy, and on researchers and public contributors themselves e.g., by gaining new knowledge and skills. Public contributors provided valuable suggestions to facilitate the involvement of people with dementia and male caregivers in future public contribution activities.

**Conclusion:**

We hope findings: (1) contribute to strengthening the evidence base on the impact of public contribution; and (2) offer insights in how to work effectively in partnership with public contributors. Future work should explore how to meaningfully involve people with dementia and male caregivers as public contributors.

